# Does the need for refinement sets of orthodontic clear aligners correlate with professional experience?

**DOI:** 10.1590/2177-6709.30.2.e2524248.oar

**Published:** 2025-05-23

**Authors:** Victor de Miranda LADEWIG, Kamila de Oliveira Novais MACHADO, Thaís Maria Freire FERNANDES, Renata Rodrigues de ALMEIDA-PEDRIN, Paula Vanessa Pedron OLTRAMARI, Márcio Rodrigues de ALMEIDA, Ana Cláudia de Castro Ferreira CONTI

**Affiliations:** 1 University of North Paraná, Dental School, Department of Orthodontics (Londrina/PR, Brazil).; 2 University of São Paulo, Dental School, Department of Orthodontics (Bauru/SP, Brasil).; 3 Anhanguera-Uniderp University, Dental School, Department of Orthodontics (Campo Grande/MS, Brazil).

**Keywords:** Orthodontics, Orthodontic appliance, Clear aligner therapy, Ortodontia, Aparelhos ortodônticos removíveis, Alinhadores estéticos transparentes

## Abstract

**Objective:**

To assess how often orthodontists request additional aligners and correlate this need with professional’s experience.

**Methods:**

A questionnaire was sent by instant messaging app (WhatsApp) to 188 orthodontists who answered it voluntarily. Respondents were grouped by experience, including time of orthodontic training (OT), training with aligners (TA) and number of cases treated with aligners (TC). They were also surveyed about the cases in which they recommend aligners. Chi-square tests were used to analyze associations between experience categories and the need for 1 or 2+ refinements, with a significance level set at 5%.

**Results:**

At least one refinement was requested in 70.31% of cases. More experienced specialists and those with longer use of aligners requested refinements more often. A second refinement was required in 21.95% of cases, primarily requested by professionals with less experience as specialists and fewer cases treated with aligners. Mild and moderate crowding were the most indicated malocclusions, while extractions and presurgical treatments were the least.

**Conclusion:**

Refinements were needed in most cases treated with aligners. Its need is not related to the lack of experience or knowledge of the technique, being mostly requested by more experienced professionals.

## RESUMO

**Objetivo:**

Avaliar com que frequência os ortodontistas solicitam alinhadores adicionais e correlacionar essa necessidade com a experiência profissional.

**Método:**

Um questionário foi enviado por um aplicativo de mensagens instantâneas para 188 ortodontistas que o responderam voluntariamente. Os respondentes foram agrupados de acordo com sua experiência, incluindo o Tempo de Formação em Ortodontia, Tempo de Trabalho com Alinhadores e Número de Casos Tratados com alinhadores. Também foram questionados sobre os casos em que indicam o uso de alinhadores. Testes qui-quadrado foram utilizados para analisar associações entre as categorias de experiência e a necessidade de 1 ou 2+ refinamentos, com nível de significância fixado em 5%.

**Resultado:**

Pelo menos um refinamento foi solicitado em 70,31% dos casos. Especialistas mais experientes e com maior tempo de uso de alinhadores solicitaram refinamentos com mais frequência. Um segundo refinamento foi necessário em 21,95% dos casos, sendo solicitado principalmente por profissionais com menos tempo de experiência como especialistas e menos casos tratados com alinhadores. Apinhamentos leves e moderados foram as más oclusões mais indicadas, enquanto extrações e tratamentos pré-cirúrgicos foram os menos indicados.

**Conclusão:**

Refinamentos foram necessários na maioria dos casos tratados com alinhadores. Sua necessidade não está relacionada à falta de experiência ou conhecimento da técnica, sendo solicitados principalmente por profissionais mais experientes.

## INTRODUCTION

Current technological advancements are changing not only how people interact with each other, but also their self-perception.^1^ ^,^ ^2^ Aesthetic appliances, once mainly a need for adult patients, are now sought by individuals of all ages.^3^ ^,^ ^4^ The rise in demand has driven both technical and technological innovations, which can now be digitally supported and fully programmed virtually.^5^ ^-^ ^10^

Aligners were initially indicated for aligning and leveling teeth, limited to small corrections^4^. However, technological advancements and the development of materials with increasingly favorable properties have gradually expanded the range of options for which aligners can be indicated.^11^ ^-^ ^14^

Despite a similar biological response, the mechanics of aligners differ from traditional fixed appliances, as they primarily “push” the tooth through elastic deformation.^15^ These unique characteristics, along with the need for patient cooperation, specific tooth movements (e.g., space closure, rotations, intrusions), and finishing/detailing, raise questions about the effectiveness of removable devices for treatment of different malocclusions.^3^ ^,^ ^7^ ^,^ ^11^ ^,^ ^14^ ^-^ ^22^

The impact of these limitations is evident in clinical practice, where only 50% of movements occur as planned.^15^ ^,^ ^21^ ^,^ ^23^ Based on the challenges of achieving specific tooth movements and the high cost of this treatment protocol, it is important to assess the need for additional aligners and identify the professional factors that contribute to this.

While some studies^20^ ^-^ ^22^ ^,^ ^24^ ^,^ ^25^ have explored clear aligner therapy (CAT) practices among orthodontists, data on the relationship between professional’s experience and the number of refinements requests remains limited. Surveys offer valuable insights,^26^ and previous studies^21^ ^,^ ^22^ using the same evaluation method (e-surveys) suggested the need for further researchers to investigate the challenges to achieve effective treatments with aligners.

Thus, the present research aimed to determine the frequency in which orthodontists need to request additional aligners during orthodontic treatment, and to correlate this need with the professional’s experience, both in terms of time and number of cases treated.

## METHODS

The research was approved by the Research Institutional Board of University of North Paraná (UNOPAR) under protocol number 42471320.4.0000.0108.

A 14-question survey, specifically designed by the authors for this study, was created using Google Forms (Google, CA, USA). Potential evaluators were digitally invited via an instant messaging app (WhatsApp, Facebook Inc, CA, USA), in groups of orthodontists from all regions of Brazil. The instrument followed the CHERRIES checklist.^27^ The first section included an explanation of the study, and participants provided consent before proceeding. The second section covered questions regarding professional location, years as an orthodontic specialist, experience with aligners, number of cases treated and refinements requested, and the types of malocclusion most commonly treated with aligners. A pilot questionnaire was conducted releasing the survey to 10 orthodontists, to ensure the instrument’s validity and reliability.

Eligible respondents included orthodontists of both genders, performing their professional activity in Brazil, who had completed at least one treatment case using esthetic aligners. The respondents were categorized based on the following criteria:

» Time of orthodontic training (OT) - years since obtaining the orthodontics specialty degree, categorized as: up to 10 years, 11 to 20 years, and over 20 years.
» Working time with aligners (TA) - years of professional experience using aligners, categorized as: up to 5 years, 6 to 10 years, and over 10 years.
» Number of treated cases (TC) - total cases treated with orthodontic aligners, categorized as: 1 to 20 cases, 21 to 50 cases, and more than 50 cases.

For evaluation purposes, professionals were instructed to consider only cases treated within the last 24 months, given the rapid technological advancements in the field in recent years that have significantly improved the clinical performance of aligners. 

### SAMPLE SIZE

The sample size calculation was based on an initial analysis of the first 100 cases, in which 76% required 1 refinement. Adopting a significance level of 5% and a statistical power of 80% to detect a difference of 10% between the groups, a minimum total sample of 321 cases per group was required. Therefore, a total of 963 cases would be necessary.

### STATISTICAL ANALYSIS

The Statistical Package for the Social Science software (SPSS, version 27.0, IBM Inc., Armonk, NY, USA) was used for data analysis. Data are presented by absolute (n) and relative (%) frequencies.

To analyze the association between the categories of orthodontists with the use of 1 refinement or 2+ refinements, the chi-square test was used. When the result was statistically significant, the comparison between the three categories was made using the proportion test, to indicate between which categories there was a statistically significant difference. The significance level in all tests was set at 5%.

## RESULTS

The survey was sent out to 350 orthodontists, and it was answered by 192. Four incomplete responses were excluded, resulting in a final sample of 188 professionals, evenly distributed by gender (96 females and 92 males). 

**Figure 1:**
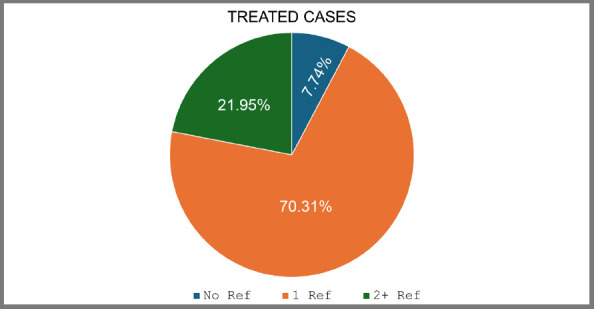
Proportion of the number of treated cases with and without refinements.

The survey reported 2,937 aligner treatments completed in the last 24 months. Of these, 70.31% required additional aligners, and 21.95% needed two or more refinements (Fig. 1).

Table 1 shows the relationship between specialization experience and the frequency of additional aligner needed over the last 24 months. Professionals with up to 10 years or 11-20 years of specialization required refinements in 72.7% and 72.9% of cases, respectively, showing no significant difference. However, those with over 20 years of experience requested refinements in 82.3% of cases. For cases requiring more than one refinement, the highest prevalence was observed among the least experienced group (27.3%), followed by those with 11-20 years (27.1%) and those with over 20 years of specialization (17.7%).

**Table 1:** Association between orthodontic training time and the use of one or more refinements.

Orthodontic training time	Participants	Cases	Ref	1 Ref		2+ Ref		p
	n	n	n	n	%	n	%	
Up to 10 years^a^	54	470	418	304	72.7	114	27.3	<0.001*
11 - 20 years^a^	90	1225	1252	913	72.9	339	27.1	
More than 20 years^b^	44	1242	1212	998	82.3	214	17.7	

*Statistically significant at p<0.05. Categories with the same superscript letters have no statistically significant difference between them. Ref = Refinement.

When considering the length of experience using aligners (Table 2), professionals with 5 years or less of experience requested refinement in 76.8% of cases, and a rate similar to 80.2% was observed in those with over 10 years of experience, with no statistically significant difference. In contrast, the group with 6-10 years of experience had the lowest refinement rate, of 71%.

**Table 2:** Association between training time with aligners and the use of one or more refinements.

Training time with aligners	Participants	Cases	With Ref	1 Ref		2+ Ref		p
	n	n	n	n	%	n	%	
Up to 5 years^a^	110	928	857	658	76.8	199	23.2	<0.001*
6 - 10 years^b^	40	649	725	515	71.0	210	29.0	
More than 10 years^a^	38	1360	1300	1042	80.2	258	19.8	

*Statistically significant at P<0.05. Categories with the same superscript letters have no statistically significant difference between them. Ref = Refinement.

Table 2 reveals that the group with 6-10 years of experience had the highest need for more than 1 refinement, of 29.0%. In comparison, professionals with over 10 years of experience using aligners requested, on average, more than one refinement in only 19.8% of cases. Those with up to 5 years of experience requested more than one refinement in 23.2% of treatments.

Table 3 shows a relationship between the number of cases treated with aligners and the need for refinement. The group that treated up to 20 cases showed the lowest refinement rate (72.4%), followed by those with more than 50 cases (76.5%) and those who treated 20-50 cases (81.0%). For cases requiring 2 or more refinements, the group with 20-50 cases had the lowest rate (19%), followed by those with more than 50 cases (23.5%) and those with 20 or fewer cases (27.6%).

**Table 3:** Association between the number of cases treated with aligners and the use of one or more refinements.

Number of cases	Participants	Cases	With Ref	1 Ref		2+ Ref		p
	n	n	n	n	%	n	%	
1 - 20^a^	85	357	337	244	72.4	93	27.6	0.012*
20 - 50^b^	52	573	520	421	81.0	99	19.0	
More than 50^ab^	51	2027	2025	1550	76.5	475	23.5	

*Statistically significant at P<0.05. Categories with the same superscript letters have no statistically significant difference between them. Ref=Refinement.

**Figure 2:**
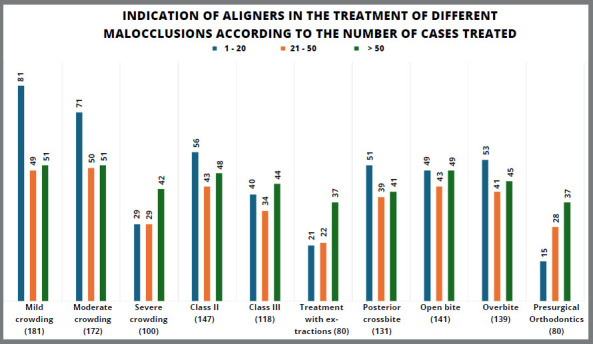
Indication of aligners in the treatment of different malocclusions according to the number of cases treated.

Figure 2 illustrates the type of malocclusions for which professionals from different groups indicated aligners as a treatment option, with mild crowding being the most common. All three groups chose aligners: 81 professionals (95.29%) in the group with up to 20 cases, 49 (94.23%) in the 21-50 cases group, and 51 (100%) in the group with over 50 cases. In contrast, presurgical orthodontics was the least commonly recommended for the up-to-20 cases group, with only 15 professionals (17.65%) selecting it. For the 21-50 cases group, aligners were least indicated for malocclusions requiring extractions, chosen by 22 professionals (42.30%). Among those with more than 50 treatments, aligners were least recommended for cases involving extractions and presurgical orthodontics, selected by 37 professionals (72.55%) in each group.

## DISCUSSION

Aligners emerge in orthodontics as highly esthetic, hygienic option, and have been increasingly requested by patients.^20^ ^,^ ^21^ ^,^ ^24^ ^,^ ^28^ However, the predictability of digitally planned tooth movements is often low, leading to the need for additional aligners, which can increase both costs and treatment duration.^7^ ^,^ ^17^ ^,^ ^23^ ^,^ ^29^ ^-^ ^31^ To assess the need for refinement sets, professionals answered a questionnaire regarding the aligner’s cases treated in the last 24 months.

Table 1 reveals that the group of professionals with over 20 years of experience in Orthodontics had the highest need for refinements (82.3%), yet also the lowest need for a second or additional refinements (17.7%). These findings align with other e-survey-based studies,^20^ ^,^ ^22^ which report that over 80% of orthodontists require refinements. Additionally, a 2019 systematic review highlighted that professional experience positively impacts the selection of appropriate techniques and the quality of treatment outcomes.^32^ A similar study comparing orthodontists and general practitioners found that specialists, being more experienced, not only take longer with virtual treatment, but are also more likely to use additional aligners to achieve the desired result.^6^

These findings may reflect the fact that more experienced professionals are more critical of treatment outcomes and strive for optimal refinement. Additionally, their advanced understanding of biomechanics and tooth movement likely explains the lower need for second refinement.

Table 2 shows that just over half of the respondents (58%) have been using aligners for less than 5 years, a finding consistent with a recent study^31^ in which 50% of respondents reported using esthetic aligners for 5 years or less. The authors noted that less experienced professionals tend to show greater interest in esthetic aligners, as the technique is commonly taught in graduate courses. In contrast, only 20.2% of respondents have used aligners for over 10 years, yet this group accounted for the highest request for additional aligners (80.2%). However, they had the lowest need for second refinements (19.8%). 

As professionals are more experienced with aligners, their confidence in treating complex cases rises,^33^ leading to longer, less predictable treatments that require more refinements. This is reflected in Table 3, in which the group with the fewest cases treated had the lowest need for additional aligners (72.4%). The next group, with 20-50 cases, despite having greater experience with aligners, required refinements in 81.0% of cases. As professionals treat more cases, they progress through the learning curve, better understanding the technique’s strengths and weaknesses. Although aligners are being indicated for increasingly complex cases, greater familiarity with the mechanics reduces the need for refinements. The group with over 50 treated cases showed a refinement rate of 76.5%, an intermediate value, compared to the two previous groups.

Mild and moderate crowding are the most commonly indicated malocclusions (Fig. 2), a finding consistent with a survey of 188 European professionals, in which crowding was the leading indication (86%).^31^ The present study shows that as crowding complexity increases, so does the number of professionals who indicate aligners. The prevalence of professionals recommending aligners for mild and moderate crowding remains similar across experience levels. However, for severe crowding, the prevalence rises with experience: 34.12% for those treating up to 20 patients, 55.77% for 21-50 cases, and 82.35% for those with over 50 cases. In other words, more experienced professionals tend to handle more complex cases.

A similar trend is seen in the malocclusions for which professionals least recommend aligners (Fig. 2). Among those with up to 20 cases, only 17.65% use aligners for pre-surgical orthodontics. For professionals with 21-50 cases, 42.30% avoid aligners for cases requiring extractions. Interestingly, while professionals with over 50 cases still use aligners less for these complex cases, their prevalence of recommendation (72.55%) is higher than that of the less experienced groups. This highlights that even for more challenging cases, experienced professionals are more likely to recommend aligners. They are better prepared to select suitable cases for aligner treatment and determine the appropriate auxiliary mechanics for each case.^6^ ^,^ ^31^

This study has limitations that must be considered. The use of aligners from different companies introduces potential bias, as variations in plastics, strength, and thickness that could affect treatment outcomes.^34^ Professional experience (years of training, number of cases, and time spent working with aligners) represents only one factor in requesting refinement sets. Other factors, including case complexity, patient compliance, and the aligner brand, also contribute to the need for refinements. Future studies should examine the impact of these variables on refinement needs.

## CONCLUSION

» Additional aligners were required in 70.31% of cases, with 21.95% needing a second refinement.
» Professionals with more specialization time requested additional aligners more often (82.3%), but had the lowest need for second refinements (17.7%).
» Orthodontists with more experience using aligners requested more refinements (80.2%) than others.
» Professionals with fewer treated cases requested the least refinements (72.4%).
» Aligners were most commonly indicated for mild and moderate crowding.

## ONLINE QUESTIONNAIRE

1. E-mail address

________________________

2. After reading the Informed Consent, I agree to participate in this research.

( ) I agree

3. Gender

( ) Male

( ) Female

4. Which state you see most of your patients?

( ) Acre

( ) Alagoas

( ) Amapá

( ) Amazonas

( ) Bahia

( ) Ceará

( ) Distrito Federal

( ) Espírito Santo

( ) Goiás

( ) Maranhão

( ) Mato Grosso

( ) Mato Grosso do Sul

( ) Minas Gerais

( ) Pará

( ) Paraíba

( ) Paraná

( ) Pernambuco

( ) Piauí

( ) Rio de Janeiro

( ) Rio Grande do Norte

( ) Rio Grande do Sul

( ) Rondônia

( ) Roraima

( ) Santa Catarina

( ) Sergipe

( ) São Paulo

( ) Tocantins

5. What year did you graduate?

________________________

6. Name of the institution you specialized in Orthodontics

________________________

7. Year you completed your specialization course

________________________

8. Which Digital Aligner Systems have you used? (Please select as many options as you wish)

In-office Aligners SHOULD NOT BE CONSIDERED.

( ) Be You

( ) Clear Aligner

( ) Clear Correct

( ) Digital Aligner

( ) E-Motion

( ) Esthetic Aligner

( ) Invisalign

( ) My Aligner

( ) Ortho Aligner (Compass)

( ) OrtoKlear

( ) Smart Aligner

( ) Unique Aligner

( ) Outro:

9. Which situation do you recommend Digital Aesthetic Aligners?

Please select as many options as you wish.

( ) Mild crowding (up to 3mm)

( ) Moderate crowding (3-7mm)

( ) Severe crowding (more than 7mm)

( ) Class II

( ) Class III

( ) Extraction treatments

( ) Posterior dentoalveolar crossbite

( ) Anterior open bite

( ) Deep overbite

( ) Pre-surgical orthodontics

10. What year did you start your first case with Digital Aesthetic Aligners?

________________________

11. Since then, how many treatments with Digital Aesthetic Aligners have you started?

( ) 1 - 5

( ) 6 - 10

( ) 10 - 20

( ) 20 - 50

( ) > 50

12. In the last 24 months, how many cases treated with Digital Aesthetic Aligners have you completed?

________________________

13. Of the cases completed in the last 24 months, how many required refinement?

________________________

14. Of the cases completed in the last 24 months, how many required MORE THAN ONE refinement request?

________________________
